# Expression of OPN3 in fibroblasts, melanocytes, and keratinocytes of skin with facial melasma in comparison with unaffected adjacent skin^☆^^☆☆^

**DOI:** 10.1016/j.abd.2020.05.016

**Published:** 2021-03-15

**Authors:** Ana Cláudia Cavalcante Espósito, Nathália Pereira de Souza, Luciane Donida Bartoli Miot, Hélio Amante Miot

**Affiliations:** Departments of Dermatology and Pathology, Faculty of Medicine, Universidade Estadual Paulista, Botucatu, SP, Brazil

Dear Editor,

Melasma is a frequent acquired chronic dyschromia that affects photoexposed areas, especially in women of childbearing age. Ultraviolet radiation (UVR) is the main environmental stimulus that induces melasma pigmentation; however, the role of visible light (VL: 400–700 nm) in its pathogenesis remains uncertain. VL comprises about 44% of solar radiation and promotes longer-lasting pigmentation (> 2 weeks) than UVA; however, only in higher phototypes (IV to VI).^1^

Opsin-3, also known as encephalopsin or panopsin (OPN3), is a G-protein-coupled photoreceptor which promotes blue light-induced melanogenesis (420–490 nm). Opsins 1 to 5 have been described in the retina; however, OPN3 is the most often expressed form in the skin, which induces the phosphorylation of the microphthalmia-associated transcription factor (MITF) in melanocytes, resulting in tyrosinase activation.^2^ To date, the differential expression of OPN3 in melasma skin compared to adjacent skin has not been investigated.

In the present study, after the Ethics Committee approval, 20 women with facial melasma (diagnosed clinically by an experienced dermatologist), without treatment for more than 30 days (except sunscreen) were submitted to two biopsies (3 mm): skin with melasma and unaffected adjacent skin (< 2 cm of distance).

The 40 specimens were submitted to triple-labeling immunofluorescence using primary antibodies and standardized dilutions: rabbit anti-OPN3 antibody (ab228748-Abcam, Cambridge-MA, USA) 1: 150; mouse anti-vimentin antibody (ab8978-Abcam, Cambridge-MA, USA) 1: 100, which is expressed in melanocytes and fibroblast intermediate filaments; and DAPI (4',6-Diamidino-2-Phenylindole), for cell nucleus labeling. Secondary antibodies (chromophores) and dilutions for OPN3: AlexaFluor 594 - 1: 500 (red); and, for vimentin: AlexaFluor 488 - 1: 500 (green). All reactions were tested against positive and negative controls.

Three areas showing greater immunolabeling in the microscopic slides in each topography (melasma and unaffected skin) were photographed using LEICA TCS-SP8 laser confocal microscopy. Using the red laser channel (OPN3 chromophore), guided by the colocalization of cytoplasmic green dots, and outside the nuclear blue colocalization, the average intensities of the image histograms (range 0 to 255) were estimated using the ImageJ software for the different cell types, blinded to the topography. The mean fluorescence intensity in each cell group was compared between the topographies by Student's *t*-test (dependent data). Cell counts between topographies were also estimated. A two-tailed p-value < 0.05 was considered significant.

OPN3 receptors were identified in all studied cells: keratinocytes, basal layer melanocytes, pendulous melanocytes, and upper dermis fibroblasts (Fig. 1). There was no differential expression in any cell group when comparing skin with melasma with the healthy adjacent skin (Table 1).

**Figure 1:**
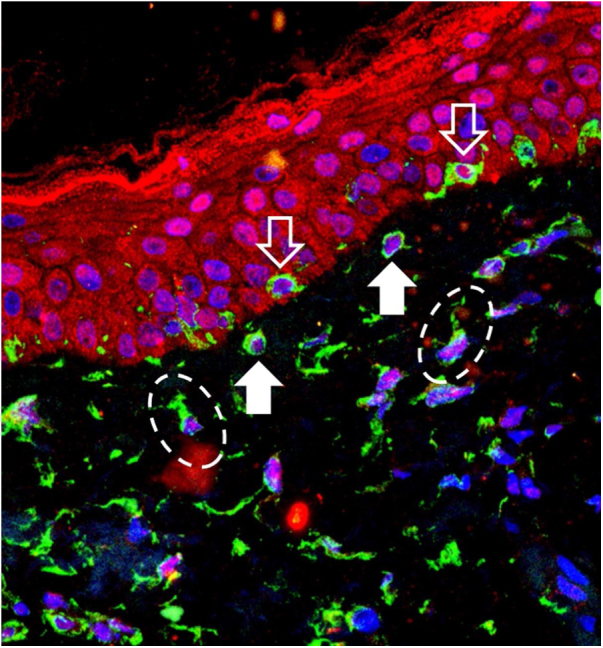
Overlapping image of triple-labeling immunofluorescence on skin with melasma. Labeling in green (vimentin), red (Opsin-3), blue (cell nuclei). White outline arrow: melanocytes in the basal layer. Full white arrow: pendulous melanocytes. Dashed ellipses: upper dermis fibroblasts.

**Table 1:** Evaluation of the mean expression (standard deviation) of OPN3 in the 20 participants, according to the topography and cell types evaluated.

	Melasma	Unaffected skin	p-value^a^
Suprabasal epidermis	30.4 (10.3)	33.9 (9.3)	0.170
Basal layer melanocytes	23.3 (9.6)	26.0 (7.9)	0.364
Pendulous melanocytes	23.7 (11.8)	22.3 (8.8)	0.652
Upper dermis fibroblasts	16.6 (10.2)	17.3 (6.7)	0.763

a Bivariate comparison between topographies.

There was no difference in melanocyte and fibroblast count in the upper dermis using a high magnification field (×400) between the two topographies. However, the skin with melasma showed a higher density of pendulous melanocytes (Table 2).

**Table 2:** Mean (standard deviation) cell counts (melanocytes, pendulous melanocytes, and fibroblasts) per high magnification field (×400).

	Melasma	Unaffected skin	p-value^a^
Basal layer melanocytes	3.2 (1.3)	3.6 (1.5)	0.451
Pendulous melanocytes	2.3 (0.9)	1.8 (0.7)	**0.031**
Fibroblasts of the upper dermis	4.5 (2.0)	4.4 (1.4)	0.915

a Bivariate comparison between topographies.

The results of this study suggest that the expression of OPN3 does not justify the difference in skin pigmentation in melasma in comparison with the unaffected adjacent skin. If the VL plays an important role in its pathogenesis, it does not play it in relation to OPN3 expression. In fact, patients with facial melasma did not show any worsening of the lesions after exposure to the computer screen (8 h/day, 5 consecutive days), at a distance of 20 cm.^3^ Moreover, the expression of opsins (OPN 1 to 5 ) in the skin is known to differ according to the phototype.^2^

There is a synergistic effect between VL and UVR type A1 (UVA1: 340–400 nm). When individuals with phototype IV to V are irradiated with isolated VL, pigmentation occurs non-linearly with the irradiated dose. When UVA1 is associated with VL, there is more intense pigmentation and the response is directly proportional to the utilized dose.^4^ The synergism of VL and UVA1 in the differential pigmentation of melasma needs to be explored using specific experimental designs. The occurrence of OPN3 in human tissues outside the retina is called “non-visual” expression, whose functions are not yet fully understood. In the skin, in addition to the modulation of melanogenesis, it promotes differentiation of keratinocytes and the activation of metalloproteinases (MMP-1, -2, -3, and -9) by fibroblasts.^2^ OPN3 can also be induced on the skin after blue light irradiation in scar tissue repair models, suggesting that it plays a role in skin healing.^5^

Pendulous melanocytes are basal layer melanocytes that protrude towards the upper dermis and are characteristic of skin with melasma, although they are found to a lesser extent in the adjacent photodamaged skin.^6^ Their pathophysiological importance is not clear, but their occurrence seems to be related to the increase in Metalloproteinase-2 and basement membrane fragmentation. Their greater numbers in the skin with melasma reinforces the role of UVR in the pathophysiology of the disease, since UVA promotes an inflammatory microenvironment in the upper dermis, with increased metalloproteinase activity (especially of MMP-2 and -9) and basement membrane degradation, which facilitates the protrusion of melanocytes towards the dermis.^7^, ^8^

This study has limitations related to the semi-quantitative *in situ* investigation technique, which does not identify different isoforms (splicing) of the photoreceptors or detects functional alterations of its pathway. There are other spindle cells in the upper dermis in addition to fibroblasts (*e.g*., CD4 + undifferentiated mesenchymal dendritic cells) which, although less numerous, are not distinguishable by morphology or vimentin labeling. Also, the other opsins (OPN-1, -2, -4, and -5) were not assessed, although less expressed in normal skin.

In conclusion, there is no differential expression of OPN3 in keratinocytes, melanocytes, or fibroblasts in facial melasma when compared to the unaffected adjacent skin.

## Financial support

FUNADERSP – *Fundo de Apoio à Dermatologia de São Paulo*.

## Authors’ contributions

Ana Cláudia Cavalcante Espósito: Participation in the design and planning of the study; collection, analysis, and interpretation of data; drafting of the manuscript; approval of the final version.

Nathália Pereira de Souza: Collection, analysis, and interpretation of data; critical review of the manuscript; approval of the final version.

Luciane Donida Bartoli Miot: Design and planning of the study; interpretation of data; critical review of the manuscript; approval of the final version.

Hélio Amante Miot: Participation in the design and planning of the study; analysis and interpretation of data; drafting of the manuscript; approval of the final version.

## Conflicts of interest

None declared.
